# Overexpression of the Potato *StPYL20* Gene Enhances Drought Resistance and Root Development in Transgenic Plants

**DOI:** 10.3390/ijms252312748

**Published:** 2024-11-27

**Authors:** Panfeng Yao, Junmei Cui, Chunli Zhang, Jia Wei, Xinglong Su, Chao Sun, Zhenzhen Bi, Zhen Liu, Jiangping Bai, Yuhui Liu

**Affiliations:** State Key Laboratory of Aridland Crop Science, College of Agronomy, Gansu Agricultural University, Lanzhou 730070, China; yaopf@gsau.edu.cn (P.Y.); cuijm@gsau.edu.cn (J.C.); zhangchunl@st.gsau.edu.cn (C.Z.); wej@st.gsau.edu.cn (J.W.); suxl@st.gsau.edu.cn (X.S.); sunc@gsau.edu.cn (C.S.); bizz@gsau.edu.cn (Z.B.); liuzhen@gsau.edu.cn (Z.L.); baijp@gsau.edu.cn (J.B.)

**Keywords:** potato, drought stress, ABA, PYR/PYL/RCAR, *StPYL20*

## Abstract

Drought is a primary limiting factor for potato growth. PYR/PYL/RCAR (referred to hereafter as PYL) proteins, as receptors for abscisic acid (ABA), play a crucial role in the plant response to drought stress. However, the underlying mechanisms of this control remain largely elusive in potatoes. In this study, a potato *StPYL20* gene was identified through genome-wide investigation and transcriptome analysis under drought stress. Molecular feature analysis revealed that the *StPYL20* gene exhibits the highest expression level in tubers, and is significantly up-regulated under ABA and drought stress conditions. The StPYL20 protein harbors a conserved domain exclusive to the PYL family. Further functional analysis showed that both transient and stable expressions of *StPYL20* in tobacco enhanced the drought resistance of transgenic plants, resulting in increased plant height, leaf number, and fresh weight, and an improved root system. Compared to wild-type plants under drought conditions, transgenic tobacco with the *StPYL20* gene exhibited lower levels of malondialdehyde (MDA), higher proline (Pro) accumulation, and increased antioxidant enzyme activity. Moreover, overexpression of the *StPYL20* gene heightened the sensitivity of transgenic plants to ABA. Furthermore, *StPYL20* up-regulated the expression of stress response and development-related genes in transgenic plants under drought stress. In conclusion, our findings indicated that *StPYL20* enhances drought resistance and root development in transgenic plants, and plays a positive regulatory role in the potato’s response to drought stress.

## 1. Introduction

The growth and development of plants are commonly influenced by a variety of abiotic stresses, such as drought, salinity, and low temperature [1]. Abiotic stress represents a primary challenge in agricultural production, significantly impacting crop yield. Following exposure to abiotic stressors, plants rapidly acclimate to adverse environmental conditions through the integration of physiological and biochemical processes, and gene regulation. Gene expression is intricately linked to stress-mediated hormonal signaling pathways, with the plant hormone abscisic acid (ABA) recognized for its pivotal role as a stress hormone in plant responses to abiotic stress [2,3].

ABA is a key phytohormone that plays a pivotal role in regulating a variety of cellular processes in plants, such as seed maturation and dormancy, seedling growth, leaf senescence, and stomatal movement [4]. Moreover, ABA plays a crucial role in mediating plant responses to a range of environmental stresses, including drought, salinity, osmotic stress, extreme temperatures, and pathogen attack [4,5]. When plants are subjected to stresses, especially dehydration stress, there is a significant increase in the level of ABA in the tissues. It has been observed that ABA can interact with the ABA receptor pyrabactin resistance 1/PYR1-like/regulatory components of ABA receptor PYL proteins. The ABA receptor PYL, protein phosphatase 2C (PP2C), and SNF1-related protein kinase 2 (SnRK2) collectively form a binary negative regulatory system PYL-PP2C-SnRK2 [6,7]. Under normal circumstances, plants exhibit low ABA levels, with PYL existing in a dimeric state unable to associate with PP2C. During this phase, PP2C demonstrates heightened phosphatase activity, impeding SnRK2 kinase activity and subsequently diminishing its capacity to phosphorylate downstream transcription factors. In response to stresses like drought, the ABA concentration rapidly escalates, binding to PYL and prompting the transformation of PYL from a dimeric configuration to a monomeric structure. The monomeric PYL form binds to PP2C alongside ABA to create a ternary complex, thereby liberating SnRK2 kinase activity to phosphorylate target proteins downstream [8,9]. Therefore, within the regulatory framework of the PYL-PP2C-SnRK2 pathway, PYL plays a crucial role at the upstream level by detecting ABA signals, inhibiting the activity of PP2C protein phosphatases, and initiating ABA signal transduction.

Since the initial identification of *PYL* in Arabidopsis, orthologous *PYL* genes have been genomically characterized in various species, with 14 PYLs in *Arabidopsis* [10], 6 *PYLs* in sweet orange [11], 13 *PYLs* in rice [12], 8 *PYLs* in grapes [13], 14 *PYLs* in rubber trees [14], 14 *PYLs* in tomato [15], 29 *PYLs* in tobacco [16], and 27 *PYLs* in cotton [17]. Recent research has also highlighted the diverse roles of *PYLs* in different species, particularly emphasizing their functions in plant development and stress responses. For instance, the overexpression of rice *OsPYL3* [18], cotton *GhPYL10/12/26* [19], or maize *ZmPYL3/9/10/13* [20] has been shown to significantly increase the sensitivity of transgenic *Arabidopsis* to ABA. Similarly, the overexpression of *OsPYL/RCAR5* or *OsPYL3/5/9/11* in rice has been linked to enhanced drought tolerance in transgenic rice plants [21,22]. Moreover, the overexpression of *OsPYL9* has been found to enhance drought tolerance and delay drought-induced leaf senescence in transgenic *Arababidopsis* and rice plants [23]. Notably, beyond herbaceous plants, the functional role of *PYL* in woody species has also been investigated. Overexpression of *PtPYRL1* or *PtPYRL5* in *Arabidopsis* and poplar has been shown to enhance ABA sensitivity and drought resistance [24]. In grapes, *VvPYL1* has been identified to interact with ABA and inhibit ABI1 phosphatase activity [25]. In recent years, many *PYL* gene families have been characterized at genome-wide levels in rice [26], grape [27], soybean [28], apple [29] and other plants [30,31]. Functional validation of the ABA receptor is pivotal for plant genetic engineering towards improving important agricultural traits such as plant biomass, yield, and tolerance to abiotic stresses. However, knowledge about *PYLs* in potato is scarce.

As the fourth largest staple crop in the world, potatoes play a crucial role in ensuring global food security [32]. Potato predominantly thrives in the northwest region of China, where the prevalent water scarcity frequently results in drought-induced stress. This phenomenon significantly impacts both local and national commodity potato production. Accordingly, it is of great importance to enhance the stress tolerance of potatoes. One of the key strategies may be achieved via genetically engineering *PYL* genes. Advances have been made in investigating key genes associated with the ABA signaling pathway in potato drought resistance. For instance, Bai et al. [33] characterized the *SnRK* gene family in potatoes, while Yao et al. [34] further confirmed the drought resistance functionality of these genes via transgenic approaches. Yao et al. [35] identified the ABA-aldehyde oxidase (AAO) gene in potatoes and conducted a screening to identify the *AAO* genes that displays a strong response to drought stress. Liu et al. [36] discovered that StHAB1 functions as a negative regulator in ABA signaling, thereby playing a crucial role in regulating potato drought tolerance and branching. Moreover, Gui et al. [37] recently discovered the *PYL* gene family in potatoes and assessed the stress responsiveness of certain genes. In this study, we identified and cloned the *StPYL20* gene. Expression analysis demonstrated that the *StPYL20* gene responds significantly to ABA treatment and drought stress. Transgenic analysis revealed that overexpression of the *StPYL20* gene markedly enhances the drought resistance of transgenic plants while also promoting root development. This study offers valuable insights for strategies aimed at enhancing tolerance to drought stress and contributes to a deeper understanding of the stress-responsive signaling networks that operate downstream of the ABA receptor.

## 2. Results

### 2.1. Cloning and Characterizations Analysis of StPYL20

**Figure 1:**
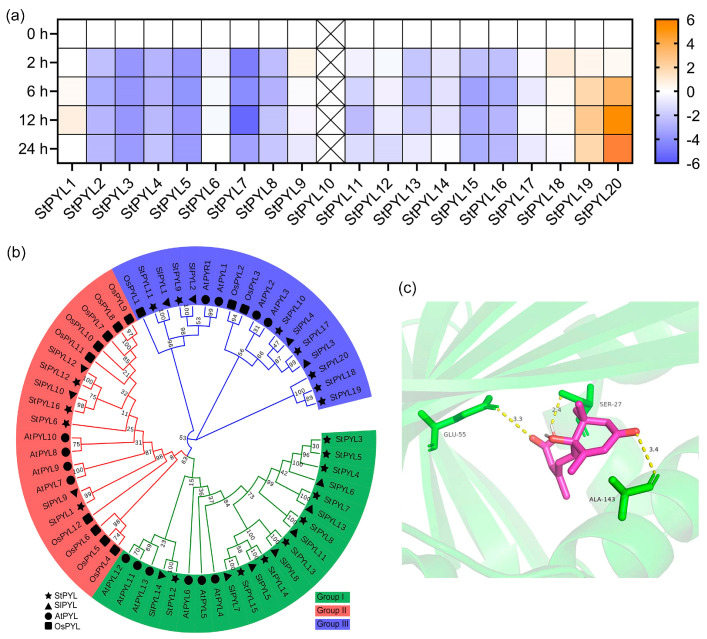
Molecular characterization of StPYL20. (**a**) Expression level analysis of *StPYLs* gene under drought stress. Three-week-old potato variety ‘QS9′ seedlings were exposed to stress treatment under simulated drought conditions using 200 mM mannitol. Whole plant samples were collected for transcriptome sequencing at 0, 2, 6, 12, and 24 h post-stress induction. The expression level of a gene at 0 h was processed as “1”, and data at other time points were normalized relative to the expression level at 0 h. The intensity of the color indicates the magnitude of the multiplier, with orange colors signifying larger multipliers and blue colors indicating smaller multipliers. (**b**) Phylogenetic relationships between StPYLs and PYLs from other plant species. St, Sl, At, and Os represent potato, tomato, Arabidopsis, and rice, respectively. Different colors represent different subfamilies. (**c**) Prediction of spatial structure depicting the binding of StPYL20 protein to ABA. The purple section illustrates the ABA molecules, while the green structural elements represent the amino acid residues in the StPYL20 protein that interact with the ABA. The yellow dashed lines symbolize the hydrogen bonds formed between residues in the StPYL20 protein and ABA. The numerical values along the dashed lines indicate the length of the hydrogen bonds.

In order to investigate the *PYL* genes in response to drought stress in potatoes, our team conducted whole genome identification of potato *PYL* family genes (Table S1). The results revealed the presence of 20 *StPYL* genes in potatoes, of which 3 *StPYLs* contain two different transcripts. Subsequently, based on the expression profiles of these *PYLs* under drought stress, we selected *StPYL20* (Accession No. XM_006345915), which significantly responded to drought stress, for functional research (Figure 1a). Further evolutionary analysis suggested that the StPYL20 protein has a relatively distant evolutionary relationship with other StPYLs (Figure 1b). Finally, a predictive binding analysis using AtPYL11 as a positive control revealed that StPYL20 exhibited stronger binding abilities to ABA, with a binding energy of −7.0, compared to the observed binding energy of −5.9 for AtPYL11. Further binding site analysis highlighted residues Glu^55^, Ser^27^, and Ala^143^ in the StPYL20 sequence as directly binding to ABA, with hydrogen bond lengths of less than 3.8, suggesting reliable results (Figure 1c).

### 2.2. StPYL20 Is Strongly Induced by ABA and Drought Treatment

**Figure 2:**
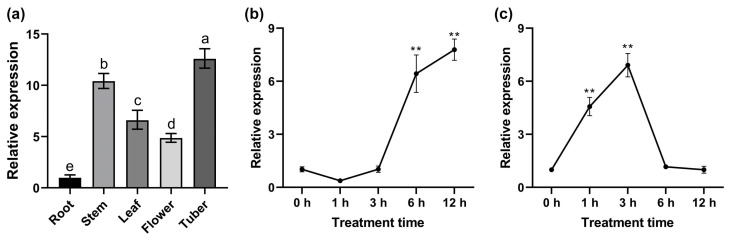
Analysis of expression level of *StPYL20* gene. (**a**) Tissue-specific expression of *StPYL20***.** Values denoted by different lowercase letters within distinct organs are significantly different (*p* < 0.05). (**b**,**c**) The expression levels of *StPYL20* under drought (**b**) and ABA (**c**) stress. Three-week-old potato seedlings were subjected to stress treatment in 1/2MS liquid medium containing a final concentration of 200 mM mannitol and 100 µM ABA. Whole plants were collected for RNA extraction after 0, 1, 3, 6 and 12 h of stress, and the expression level of *StPYL20* was detected by qRT-PCR. The 2^−ΔΔCT^ method was used to evaluate the relative expression, and the expression levels of genes in root tissue and stress for 0 h were defined as “1”. Mean values from three independent biological replicates, each with 9 plants from each line, +/− SD, are shown. Statistical significance of differences between groups was determined by one-way ANOVA (with Tukey’s test). ** indicates significant difference at *p* < 0.01 level compared to the control group (0 h).

To examine the expression profile of *StPYL20* across various tissues, qRT-PCR was conducted using mRNA samples from distinct tissue types. The findings revealed substantial variances in *StPYL20* expression levels across all examined tissues, with statistically significant disparities observed between tissues. Among them, *StPYL20* has the highest expression level in tubers, which is 12.5-, 1.2-, 1.9-, and 2.6-fold higher than in roots, stems, leaves, and flowers, respectively (Figure 2a). Furthermore, we conducted a detailed analysis of the impact of ABA and drought stress on the transcription levels of *StPYL20*. Following exposure to drought stress, the expression levels of *StPYL20* exhibited no significant alteration within the initial 3 h period, subsequently recorded a significant upsurge after 6 h, and reached its maximum after 12 h (Figure 2b). In contrast, the response pattern of *StPYL20* to ABA differed noticeably. The expression levels of *StPYL20* experienced a substantial increase after 1 h of ABA stress, reached its zenith after 3 h, and then receded to a level insignificantly different from the baseline (0 h) measurement (Figure 2c).

### 2.3. Transient Transformation of StPYL20 Enhances the Drought Tolerance of Transgenic Tobacco

**Figure 3:**
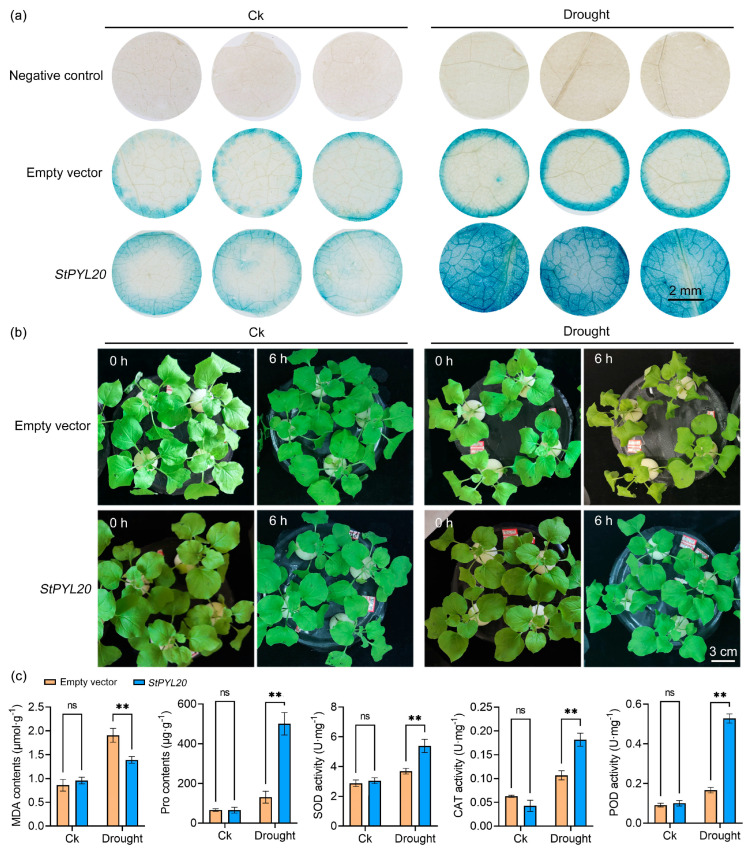
Identification of drought resistance in tobacco after transient transformation of *StPYL20* gene. Transferring 3-week-old tobacco seedlings to Hoagland solution for normal cultivation for 2 days, the transient expression of the *StPYL20* gene was performed. Two days after transient expression, samples were collected for GUS staining to confirm successful transformation of the target gene. In the drought stress experiment, tobacco plants with successful transient expression were transferred to Hoagland nutrient solution containing 200 mM mannitol for stress induction. Leaf samples were collected 6 h after treatment to measure various physiological parameters. Tobacco plants cultured in normal Hoagland nutrient solution were used as a control (CK). (**a**) GUS histochemical staining of *StPYL20* transgenic plants under normal conditions and drought stress; (**b**) phenotypic collection of each genotype before and after drought stress; (**c**) determination of physiological indexes related to stress. Mean values from three independent biological replicates, each with 9 plants from each line, +/− SD, are shown. Statistical significance of differences between groups was determined by one-way ANOVA (with Tukey’s test). ** indicates a significant difference at *p* < 0.01 levels. ns indicates that the difference is not significant.

To preliminarily assess the functionality of *StPYL20*, we first conducted a transiently overexpression of it in tobacco and subjected plants to drought treatment to investigate its potential impact on the drought resistance of transgenic plants. The schematic diagram of the *StPYL20* gene plant overexpression vector is shown in Figure S1. Initially, GUS staining results indicated successful integration of the target gene, as evidenced by a prominent blue coloration in the leaves of both empty vector and *StPYL20*-transformed plants. Subsequently, it was observed that under drought conditions, the blue hue in the leaves of *StPYL20* transgenic plants was notably more intense compared to the control group (Figure 3a). Further phenotypic evaluations revealed that the overexpression of *StPYL20* ameliorated the wilting symptoms in transgenic plants exposed to drought stress, suggesting that the expression of *StPYL20* bolstered the drought tolerance of tobacco (Figure 3b).

Furthermore, in order to elucidate the effects of *StPYL20* on the physiological status of transgenic plants, we assessed the accumulation of malondialdehyde (MDA) and proline (Pro) closely associated with plant stress responses, as well as the activities of antioxidant enzymes such as superoxide dismutase (SOD), peroxidase (POD), and catalase (CAT). The results revealed that overexpression of *StPYL20* led to a notable increase in Pro accumulation while decreasing MDA levels in transgenic plants subjected to drought stress. The activities of the three antioxidant enzymes exhibited a similar pattern of change. Specifically, under normal conditions, there was no significant disparity in enzyme activity between transgenic and control plants. However, under drought stress conditions, the enzyme activity in transgenic plants was markedly higher than that in control plants (Figure 3c).

### 2.4. Generation of Stable Transgenic Tobacco

To enhance the understanding of the drought resistance mechanism of *StPYL20*, we established stable expression of *StPYL20* in tobacco via the *Agrobacterium*-mediated leaf disc transformation method. Nine transgenic plant lines exhibiting resistance to hygromycin were further subjected to GUS staining, PCR identification targeting the hygromycin gene, and qRT-PCR analysis targeting the *StPYL20* gene (Figure S2). Following this, three transgenic lines (OE-5, OE-13 and OE-18) with high levels of *StPYL20* expression were selected for subsequent investigations.

### 2.5. Overexpression of StPYL20 Increases Tobacco Drought Tolerance

**Figure 4:**
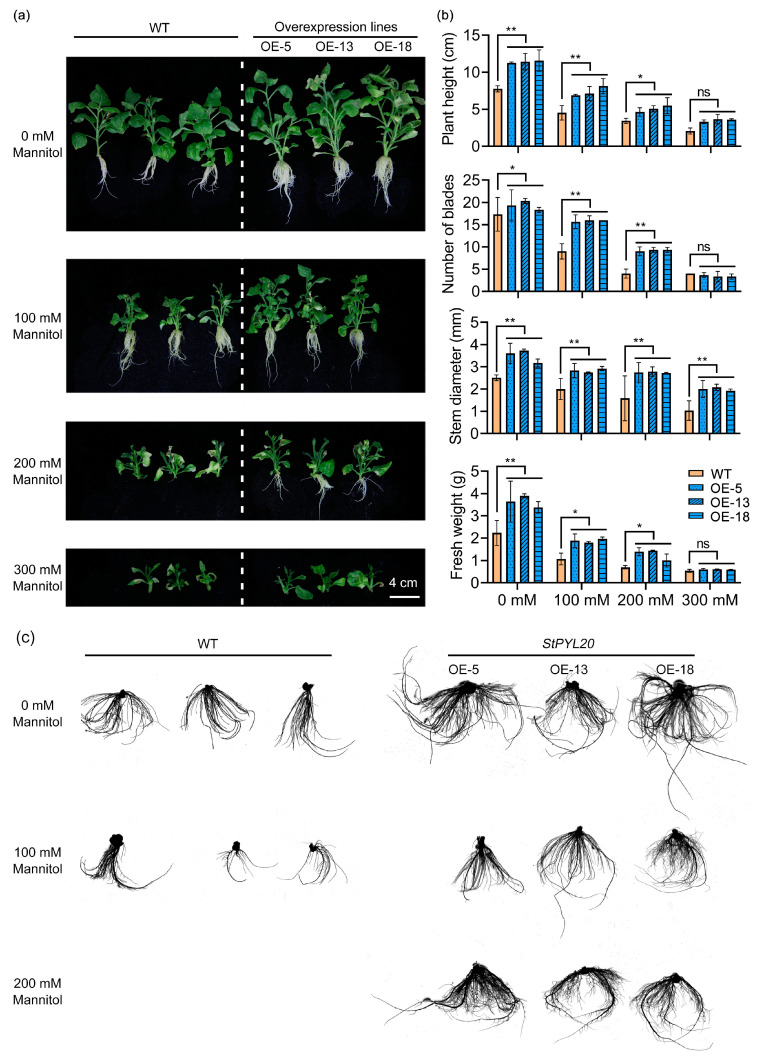
Evaluation of drought resistance in *StPYL20* transgenic plants. Transgenic tobacco stem segments displaying consistent growth were cultured on MS medium, with additional treatments of 100 mM, 200 mM, and 300 mM mannitol for 30 days to induce stress, after which various phenotypic traits were assessed: (**a**) the phenotypic characterization of plants under drought conditions; (**b**) quantification of plant height, leaf number, stem diameter, and fresh weight under both normal and drought stress conditions; (**c**) root scanning diagram of each plant under different treatment conditions. After 30 days of growth under normal and stress conditions, plant root systems were washed with water to remove the culture medium, followed by scanning of the roots using a root scanner (LD-WinRHIZO). Mean values from three independent biological replicates, each with 9 plants from each line, +/− SD, are shown. Statistical significance of differences between groups was determined by one-way ANOVA (with Tukey’s test). *, ** denote statistical significance at *p* < 0.05 and *p* < 0.01 levels, respectively. ‘ns’ signifies no significant difference. A horizontal line above columns indicates data points with similar levels of significance compared to the wild-type control.

To evaluate the drought resistance function of *StPYL20*, this study subjected transgenic tobacco and control plants to three distinct drought conditions and assessed their phenotypic responses after a 30-day stress period through photographic documentation (Figure 4a). Results revealed pronounced variations in growth parameters between transgenic and control plants under normal conditions, with transgenic specimens displaying superior plant height, leaf count, stem diameter, and fresh weight compared to controls (Figure 4b). Upon exposure to stress, growth inhibition was observed across all plants, with control specimens experiencing more pronounced stunting relative to transgenic counterparts. This difference was exacerbated with escalating stress intensity. Notably, control plants exhibited complete root growth suppression under stress induced by 200 mM mannitol, whereas transgenic plants maintained regular root development. At 300 mM mannitol stress levels, all plants showed complete growth inhibition, with no discernible disparities in plant morphology or associated phenotypic traits.

During the phenotype collection process, it was observed that the root systems of transgenic plants exhibited greater luxuriance compared to those of control plants under both normal and stress conditions. Subsequently, a detailed analysis of the root systems of each plant was conducted. Morphologically, *StPYL20* transgenic plants displayed enhanced root development in comparison to control plants under normal as well as stress conditions (Figure 4c). These findings were further substantiated through the assessment of relevant parameters. Specifically, key indicators such as total root length, root area, root forks, and total root volume demonstrated consistent patterns of alteration. Notably, it was observed that the root diameter remained relatively unchanged before and after exposure to stress during normal root growth (Figure S3).

**Figure 5:**
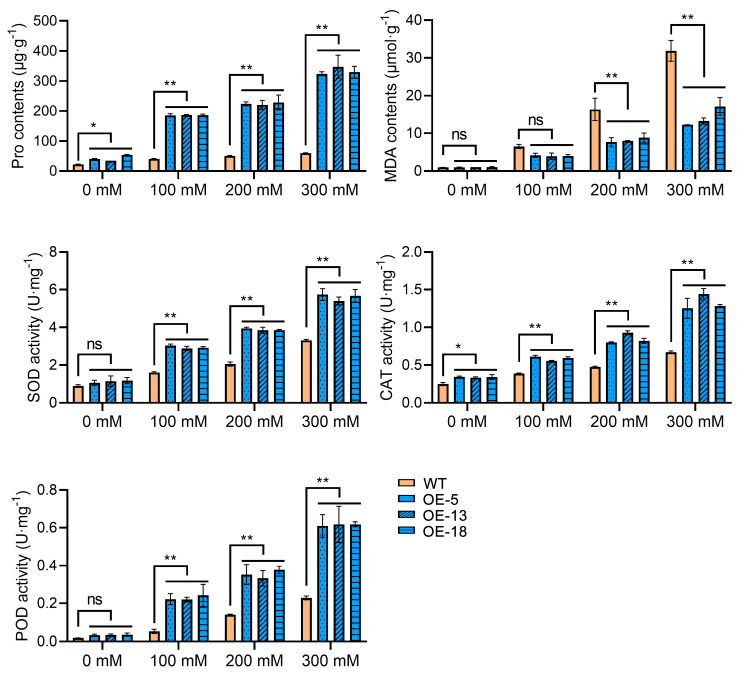
Determination of stress-related physiological index contents in different plants under different treatment conditions. The physiological indicators mainly include Pro content, MDA content, and antioxidant enzyme activity. Mean values from three independent biological replicates, each with 9 plants from each line, +/− SD, are shown. Statistical significance of differences between groups was determined by one-way ANOVA (with Tukey’s test). *, ** denote statistical significance at *p* < 0.05 and *p* < 0.01 levels, respectively. ‘ns’ signifies no significant difference. A horizontal line above columns indicates data points with similar levels of significance compared to the wild-type control.

Additionally, two key physiological indicators closely associated with drought stress, Pro and MDA, were assessed. The accumulation of Pro in transgenic plants was significantly higher than that in control plants across all conditions, with the disparity escalating as treatment conditions intensified. MDA content exhibited a notable variance between the 200 mM and 300 mM mannitol conditions, whereas no significant distinction was observed between the strains under normal circumstances and the 100 mM mannitol conditions. Subsequently, the activities of three antioxidant enzymes pertaining to antioxidant efficacy were further elucidated. SOD and POD activities displayed a corresponding pattern of alteration. Under normal conditions, no significant differences were observed between transgenic and control plants. However, under stress conditions, the enzyme activity in transgenic plants exceeded that of control plants, with the disparity becoming more pronounced as the severity of the treatment conditions increased. Notably, CAT activity in transgenic plants exceeded that in control plants throughout all conditions, with the discrepancy peaking under the 300 mM mannitol conditions (Figure 5).

### 2.6. Overexpression of StPYL20 Increases the Sensitivity of Transgenic Plants to ABA

**Figure 6:**
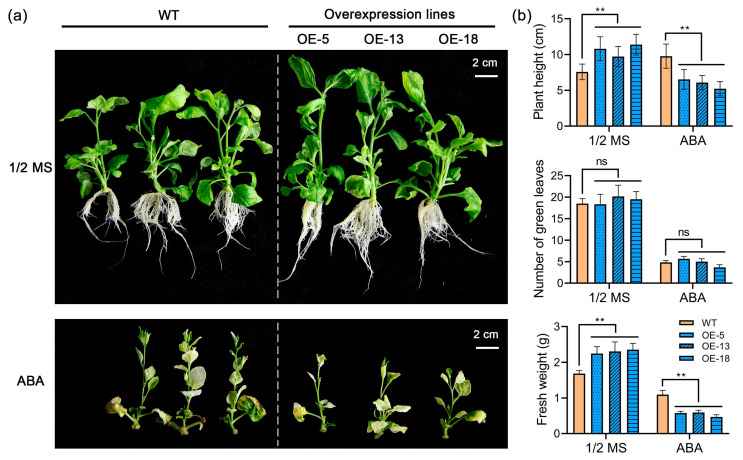
Detection of ABA sensitivity in transgenic plants overexpressing *StPYL20*. Transgenic tobacco stem segments exhibiting consistent growth were cultured on 1/2MS medium alone or supplemented with 20 µM ABA to induce stress, following which various phenotypic characteristics were evaluated. (**a**) Phenotypic profiling of plants under control and stress conditions; (**b**) quantification of plant height, number of green leaves, and fresh weight under both control and drought stress conditions. Mean values from three independent biological replicates, each with 9 plants from each line, +/− SD, are shown. Statistical significance of differences between groups was determined by one-way ANOVA (with Tukey’s test). ** indicates statistical significance at *p* < 0.01 level. ‘ns’ denotes no statistically significant difference. A horizontal line above columns indicates data points sharing similar levels of statistical significance compared to the wild-type control.

We further investigated the impact of *StPYL20* overexpression on the sensitivity of transgenic plants to ABA. Under normal conditions, phenotypic observations and measurements indicate that overexpression of *StPYL20* notably enhances the plant height. When exposed to ABA stress, transgenic plants exhibited heightened sensitivity compared to the control (Figure 6a). Despite growth inhibition in all strains, transgenic plants experienced more pronounced inhibition, resulting in significantly lower plant height and fresh weight than control plants. Furthermore, statistical analysis of green leaf count revealed no significant difference between transgenic plants and the control group (Figure 6b).

### 2.7. Expression of Drought Response and Development Related Genes in Transgenic Tobacco

**Figure 7:**
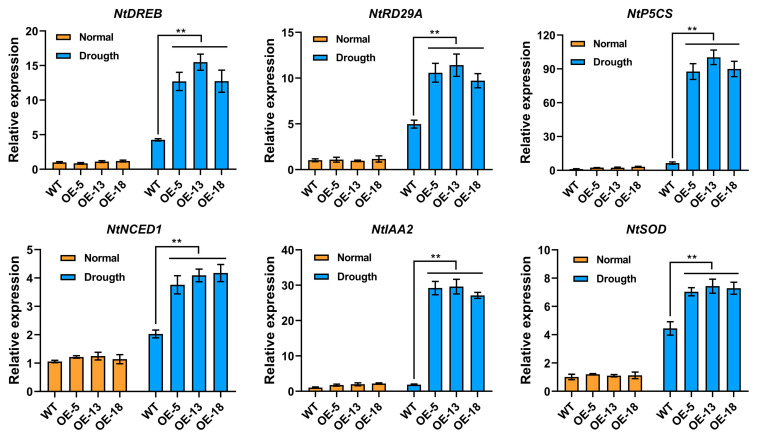
Analysis of stress-responsive genes in transgenic and WT plants under normal and drought stress. The total RNA was isolated from transgenic and wild-type tobacco after 30 days under 100 mM mannitol treatments. The 2^−ΔΔCT^ method was used to evaluate the relative expression, and the expression levels of genes in the WT plants under normal conditions were defined as “1”. Mean values from three independent biological replicates, each with 9 plants from each line, +/− SD, are shown. Statistical significance of differences between groups was determined by one-way ANOVA (with Tukey’s test). **, denote statistically significant differences at *p* < 0.05 levels.

To further investigate the potential mechanism by which *StPYL20* enhances drought tolerance in transgenic plants, we conducted an analysis of the expression patterns of multiple genes across various strains under both normal and drought conditions (Figure 7). The genes examined included those related to stress response (*NtDREB*, *NtRD29A*), proline biosynthesis (*NtP5CS*), ROS scavenging (*NtSOD*), auxin signaling (*NtIAA2*), and ABA biosynthesis (*NtNCED1*). Our results indicated that, under normal conditions, the overexpression of the *StPYL20* gene did not lead to significant changes in the expression levels of the analyzed genes. However, under drought stress, the expression levels of these genes in transgenic plants were markedly elevated compared to those in control plants. Notably, *NtIAA2* and *NtP5CS* exhibited the most pronounced increases, with expression levels reaching 15.2- and 14.3-fold higher than those of the control, respectively. These findings suggested that the overexpression of *StPYL20* may play a direct or indirect role in regulating the expression of these genes, thereby contributing to enhanced drought resistance in plants.

## 3. Discussion

The plant hormone ABA is integral to the regulation of plant growth, development, and stress responses [38]. Drought stress typically triggers an increase in intracellular ABA synthesis, with the ABA receptor protein PYL playing a pivotal role in the ABA-mediated signaling pathway that governs plant mechanisms for drought resistance [15]. While substantial research has focused on the function of PYL proteins in enhancing drought tolerance across various plant species, investigations into the specific role of PYL proteins in response to drought stress in potatoes remain limited. To investigate the *PYL* genes involved in drought stress response in potatoes, our research team conducted a comprehensive genome-wide identification of the potato *PYL* gene family three years ago. The results indicated the presence of 20 *PYL* genes in the potato genome, with 3 of these genes exhibiting two distinct transcripts. Subsequently, based on the expression profiles of these *PYL* genes under drought stress conditions, we selected *StPYL20*, which demonstrated a significant response to drought stress, for further functional analysis. However, GUI et al. [37] first published an article on the identification of the potato *PYL* gene family two months ago. Compared with the results of GUI et al. [37], we have identified three new *StPYL* genes, named *StPYL18*, *StPYL19*, and *StPYL20*. Further protein structure analysis showed that the proteins encoded by these three PYL genes contain the Pfam: Bet-v_1 conserved domain unique to the PYL family (Figure S4). Homology alignment in the Arabidopsis TAIR database indicated that these three StPYLs exhibit high similarity with AtPYL11 and AtPYL12, respectively. Further evolutionary analysis suggested that these three StPYLs proteins have a relatively distant evolutionary relationship with other StPYLs (Figure 2), potentially explaining why they were excluded by Gui et al. Finally, a predictive binding analysis using AtPYL11 as a positive control revealed that the three StPYLs exhibited stronger binding abilities to ABA with binding energies of −8.1 (StPYL18), −6.3 (StPYL19), and −7.0 (StPYL20), compared to −5.9 observed for AtPYL11. Following comprehensive molecular characterization, we confirmed the regulatory effect of *StPYL20* on drought resistance by evaluating transient and stable expression in transgenic tobacco plants.

With the rapid advancements in gene sequencing technologies, an increasing number of plant genomes have been sequenced, facilitating the screening and identification of gene families and key genes that are essential for plant growth and stress regulation at a whole-genome level [39]. Although ABA has a critical role in this context, it was not until 2009 that scientists identified the ABA receptor protein PYL [10]. This significant discovery has prompted a growing body of research focused on the screening and identification of PYL family proteins across various plant species [40]. For instance, in *Arabidopsis*, 14 PYL proteins have been classified into three subfamilies based on phylogenetic analysis, as well as structural and functional characterization [41]. Whole-genome screenings of plant PYL family proteins have revealed notable differences in the number of PYL family genes among different plant species. Specifically, poplar contains *14 PYL* genes [16], upland cotton has 40 [17], tomato possesses 15 [15], grape contains 8 [27], wheat contains 38 [42], and rice has 13 [26]. Despite these significant variations in the number of *PYL* family genes, the clustering patterns of these genes remain consistent across plant species. The *PYL* family members within these species are categorized into three subfamilies [26]. Recent studies have identified the molecular characteristics of the potato *PYL* gene family through comprehensive genomic analysis. Subsequently, the response of various *StPYL* genes to drought stress was evaluated, indicating that the *StPYL20* gene showed a significantly high response under ABA and drought conditions. This discovery suggests that the *StPYL20* gene may play a key role in the potato’s ability to cope with drought stress.

Drought, acknowledged as a significant abiotic stress factor, poses a considerable threat to global agricultural production, leading to a pronounced decrease in crop yields. In the realm of plant responses to abiotic stresses like drought, several plant stress hormones, with ABA notably, assume a crucial regulatory role [43]. Notably, PYL proteins, which serve as the primary receptors for ABA, are essential for the perception of stress signals and their subsequent downstream effects. A wealth of studies has demonstrated that PYL proteins significantly enhance plant drought tolerance. For instance, overexpression of the *AtPYR1* and *AtPYL1/2/3/8/9* genes has been shown to significantly enhance drought tolerance in transgenic plants [44]. Similarly, comparable results have been observed in crops such as maize [20], wheat [45], rice [18], poplar [24], and cotton [19]. Specifically, the *PYL4* gene in wheat has been found to improve water use efficiency and increase drought resistance by reducing stomatal opening and enhancing photosynthesis in transgenic plants. Furthermore, overexpression of rice *PYL3/10* [18] and cotton *PYL10/12/26* [19] enhances seed sensitivity to ABA during germination and seedling stages, thereby improving adaptability to abiotic stress. Additionally, *PYL3/9/10/13* in maize has been shown to increase drought tolerance in transgenic plants by promoting the accumulation of proline under drought stress conditions [20]. Research on the *PYL* gene family in crops such as grape [27], tomato [46], canola [47], strawberry [48], and apple [49] indicates that modulating the expression levels of these genes can enhance plant resistance to abiotic stress. In this study, transcriptomic analysis under early drought stress conditions revealed the involvement of the *PYL* gene family member *StPYL20* in potato’s response to drought stress. Subsequent confirmation through transgenic technology demonstrated the positive regulatory role of *StPYL20* in drought stress response. Similar to previous studies on the function of *PYL* genes, overexpression of *StPYL20* mitigated the detrimental effects of drought stress on transgenic plants and promoted their growth under stress conditions. Moreover, the analysis of the response to ABA indicated that the expression of the *StPYL20* gene significantly increases one hour after stress exposure, subsequently returning to baseline levels after 6 h. These findings suggested that *StPYL20* may be induced by ABA and thus play a role in regulating responses to abiotic stress. Further investigations revealed that the overexpression of *StPYL20* markedly enhances the sensitivity of transgenic plants to ABA. Under ABA treatment, the growth of the transgenic plants is significantly inhibited. Therefore, it can be inferred that *StPYL20* enhances ABA signal sensitivity, effectively activating downstream adverse response signaling pathways, thereby improving the drought resistance of the transgenic plants.

When plants experience drought stress, the balance between the generation and scavenging of ROS is rapidly disrupted, leading to an accumulation of ROS, which in turn triggers oxidative damage and ultimately results in cell death [50]. This phenomenon has been extensively studied and confirmed in various plant species. In response to drought stress, plants typically up-regulate the transcription levels of antioxidant enzyme genes such as SOD, POD, and CAT, thereby enhancing the activity of these enzymes to promote the scavenging of ROS [51,52]. These mechanisms are crucial for improving the adaptability of plants under drought stress. Our study indicates that under drought stress conditions, the expression levels of the *SOD* gene and its enzymatic activity are significantly elevated in *StPYL20* transgenic plants compared to control plants. Additionally, the reduction in MDA content in the transgenic plants further supports this observation, indicating a decrease in oxidative damage. The ROS scavenging system plays a critical role in helping plants withstand drought stress, comprising various enzymes and non-enzymatic scavengers. These enzymes work collaboratively to mitigate the harmful effects of ROS [53,54]. MDA, a product of lipid oxidation, is widely regarded as a marker of oxidative damage in plants [55]. Physiological results indicated that the *StPYL20* transgene did not influence ROS accumulation under control conditions. However, under drought stress conditions, the ROS content significantly decreased, while the activities of CAT, POD, and SOD were significantly elevated in *StPYL20* transgenic plants compared to WT plants. Given that drought treatment is known to increase ABA levels, we hypothesize that augmented ABA binds to the elevated StPYL20, thereby activating downstream stress-responsive pathways and regulating ROS metabolism. Further investigations are necessary to elucidate the mechanisms by which ABA receptors modulate ROS metabolism. Pro, an osmotic substance present in plant cytoplasm, plays a key role in maintaining the structural integrity of cell membranes and proteins, scavenging ROS, and minimizing light-induced damage to chloroplast thylakoid membranes [56]. In this study, the overexpression of *StPYL20* resulted in a significant increase in free proline content in transgenic plants under drought stress conditions. This change was accompanied by the up-regulation of the key gene *NtP5CS*, which is involved in proline synthesis. The biosynthesis of proline in plants primarily occurs through two pathways: the glutamate pathway and the ornithine pathway, with the glutamate pathway being the most active under osmotic stress conditions. In the glutamate pathway, glutamate (Glu) is converted into gamma-glutamyl semialdehyde (GSA) by the enzyme pyrroline-5-carboxylate synthetase (P5CS). Subsequently, GSA spontaneously cyclizes to form pyrroline-5-carboxylic acid (P5C). Following this, P5C is catalyzed by pyrroline-5-carboxylate reductase (P5CR) to yield proline [57]. The P5CS gene serves as a key rate-limiting enzyme in this biosynthetic pathway and is critical for proline biosynthesis [58]. Consequently, the results of this study suggested that *StPYL20* may enhance drought resistance in transgenic plants by regulating proline synthesis. This process warrants further experimental investigation to elucidate its molecular mechanisms.

Furthermore, previous studies have indicated that *PYL* genes play a crucial role in plant growth and development, potentially enhancing drought resistance by regulating these processes. For instance, it has been shown that the overexpression of the *GhPYL10/12/26* genes in cotton can improve drought tolerance in transgenic plants and promote root enhancement under normal growth conditions [19]. In *Arabidopsis*, *PYL9* not only enhances drought resistance by reducing water evaporation but also promotes senescence in older leaves while inhibiting the growth of young tissues in response to severe drought stress [23]. Notably, this study has observed similar findings. Specifically, under normal growth conditions, transgenic plants with the *StPYL20* gene exhibited superior growth and more developed root systems compared to control plants. Additionally, under drought conditions, the expression level of *IAA2* in transgenic plants was approximately 30 times higher than that in control plants, suggesting that the up-regulation of *StPYL20* not only enhances drought resistance through proline synthesis but also contributes to the regulation of plant growth and development. The up-regulation of the *StPYL20* gene led to the development of robust root structures in transgenic plants, increasing their ability to absorb water and thereby enhancing drought tolerance. Plants that are tolerant to abiotic stress or exhibit hypersensitivity to abscisic acid (ABA) may experience growth retardation due to their heightened sensitivity in perceiving and responding to environmental stresses. This increased sensitivity often leads to the diversion of resources towards stress protection mechanisms rather than promoting growth or yield. Consequently, it is a widespread issue that genes associated with enhancing abiotic stress tolerance can result in growth inhibition, even under optimal growth conditions, when these genes are constitutively overexpressed. Notably, the constitutive overexpression of genes related to ABA signaling can markedly impede growth, as ABA plays a central role in regulating both plant development and growth processes [11]. For example, transgenic plants overexpressing the *Arabidopsis* down-regulating *β*-subunit of farnesyltransferase (AtFTB), a negative regulator of ABA signaling, are drought-tolerant but also show seedling growth inhibition [59]. This indicates that the regulation of plant growth, development, and response to environmental stresses is a highly intricate physiological process, characterized by significant variations among different species and gene families. This study is the first to investigate the function of the potato ABA receptor protein PYL under both ABA treatment and drought stress, thereby establishing a robust foundation for understanding the ABA signaling pathway in potatoes in response to drought conditions. Moving forward, we aim to broaden our research by screening for interacting proteins of StPYL20 under drought stress and investigating the transcriptional changes in transgenic plants. Through a comprehensive approach that integrates physiological, biochemical, transcriptomic, and proteomic methodologies, we will elucidate the molecular mechanisms through which the *StPYL20* gene contributes to plant drought resistance.

## 4. Materials and Methods

### 4.1. Identification StPYL Genes

The potato reference genome sequence and GFF annotation file were acquired from the potato genome website (http://spuddb.uga.edu/, accessed on 10 January 2022), while all AtPYL protein sequences were sourced from TAIR (https://www.arabidopsis.org/, accessed on 13 March 2022). The StPYL protein sequences were derived through sequence alignment and screening using TBtools (v2.136). Subsequently, the obtained StPYL protein sequences were subjected to comparison in Pfam (http://pfam.xfam.org/, accessed on 13 April 2022), SMART (http://smart.embl-heidelberg.de/smart/batch.pl, accessed on 20 April 2022), Ensembl Plants (http://plants.ensembl.org/index.html, accessed on 20 April 2022), and NCBI websites (http://spuddb.uga.edu/, accessed on 23 April 2022), with target sequences being annotated to ascertain the definitive potato StPYL proteins.

The molecular characteristics of the *StPYL* gene were further analyzed. The NCBI Batch CD search online website (https://www.ncbi.nlm.nih.gov/Structure/bwrpsb/bwrpsb.cgi, accessed on 8 May 2022) was utilized to analyze the conserved structural domains of the PYL proteins, followed by visualization using TBtools software. Additionally, the PYL protein sequences of *Arabidopsis thaliana*, *Oryza sativa*, and *Solanum lycopersicum* were downloaded from NCBI. Subsequently, a phylogenetic tree including the StPYL protein and the aforementioned three species was constructed using MEGA7 software (V7.0.26). Finally, the phylogenetic tree was visually enhanced through the EvolView online platform (https://evolgenius.info//evolview-v2/#login, accessed on 27 July 2022).

### 4.2. Plant Growth

The drought-resistant potato variety Qingshu 9 (QS9) was cultivated and provided by the Biotechnology Research Institute of the Qinghai Academy of Agricultural and Forestry Sciences, with its parental lines being CIP387521.3 and CIPAPHRODITE. The tobacco seeds were preserved at the State Key Laboratory of Aridland Crop Science, Gansu Agricultural University. In the absence of special instructions, potato and tobacco seedlings were cultured in an artificial growth chamber under a light regimen of 16 h of illumination followed by an 8-h dark period, maintaining a temperature of 22 ± 2 °C and a relative humidity of 60%.

### 4.3. Evaluation of Drought Resistance After Transient Transformation of Tobacco

The open reading frame (ORF) of *StPYL20* was amplified and digested with *Bgl*II and *Spe*I enzymes, then inserted into the vector 35S-1304-GUS using the homologous recombination method. Subsequently, the recombinant plasmid 35S-StPYL20-GUS was introduced into *Agrobacterium tumefaciens* GV3101. Three-week-old tobacco seedlings were transplanted into Hoagland solution, and after a two-day incubation period, they were employed for *StPYL20* transient expression mediated by GV3101. Two days post-instantaneous expression, samples were collected for GUS staining to confirm the successful transformation of the target gene. In the drought stress experiment, tobacco plants that had successfully undergone transient expression were transferred to Hoagland nutrient solution containing 200 mM mannitol for stress induction. After 6 h of stress treatment, leaf samples were collected to assess various physiological parameters associated with drought stress. Tobacco materials cultured under normal Hoagland conditions were used as a control.

### 4.4. Evaluation of Drought Resistance After Stable Transformation of Tobacco

The plasmids used in the stable expression assay were constructed by ligating the ORF sequence of *StPYL20* with pCAMBIA1301 using *Kpn*I and *BamH*I. The recombinant plasmids were transformed into *Nicotiana tabacum* (T12) by utilizing the *Agrobacterium*-mediated leaf disk transformation method [60]. After callus differentiation into seedlings, transgenic plants were subjected to resistance screening on 1/2 MS medium containing hygromycin (50 mg L^−1^, *w*/*v*). Subsequently, positive lines were identified through RT-PCR with the tag gene on vector and GUS histochemical staining. Finally, the top three transgenic lines with the highest expression levels of *StPYL20* were selected based on qRT-PCR. To induce ABA and drought stress, the transgenic lines and wild-type plants were cut into uniform stem segments and exposed to stress treatment on 1/2 MS medium supplemented with 20 mM ABA, as well as 100 mM, 200 mM, and 300 mM mannitol. The normal 1/2 MS medium served as the control. Following a 30-day stress duration, the physiological markers associated with stress were assessed.

### 4.5. Determination of Phenotypic and Physiological Indicators

The phenotypic traits encompassed plant height, leaf number, stem diameter, and fresh weight. Additionally, the total root length, number of root tips, root diameter, total root area, and total root volume were also assessed utilizing a root scanning device. The levels of MDA and Pro, alongside the activity of SOD, POD, and CAT, were quantified as established protocols [61].

### 4.6. Histochemical Staining of GUS

The GUS staining process followed the previous method [62]. Leaves were removed and placed in a solution containing 2 mM 5-bromo-4-chloro-3-indolyl-beta-d-glucuronic acid, 0.1 M sodium phosphate buffer (pH = 7.0), 0.5 mM of potassium ferrocyanide each, 10 mM EDTA (pH = 7.0), and 0.10% Triton X-100 at 37 °C for 12 h. After eliminating chlorophyll with 70% ethanol washes, GUS staining results were observed and photographed.

### 4.7. Expression Measurement of Stress-Responsive Genes

The RNA was extracted from tobacco leaves and converted into cDNA through reverse transcription. The expression of stress-related genes was then evaluated via qRT-PCR using specific primers listed in Table S2.

### 4.8. Statistical Analysis

The data, derived from three biological replicates, are presented as means ± SD. Statistical analysis was performed using one-way analysis of variance (ANOVA), followed by Tukey’s post hoc tests for multiple comparisons. Statistical significance was defined at *p* < 0.05 or *p* < 0.01.

## 5. Conclusions

This study demonstrates that the transient and stable overexpression of the *StPYL20* gene in tobacco enhances root development and promotes the expression of stress-related genes, antioxidant enzyme genes, and ABA biosynthesis genes. Consequently, this leads to increased sensitivity to ABA and positively regulates drought stress responses.

## Data Availability

The original contributions presented in this study are included in the article/supplementary material. Further inquiries can be directed to the corresponding author.

## Supplementary Materials

The following supporting information can be downloaded at: https://www.mdpi.com/article/10.3390/ijms252312748/s1.
