# Supplementary material for: Overexpression of the Potato StPYL20 Gene Enhances Drought Resistance and Root Development in Transgenic Plants
Source: Int J Mol Sci. 2024 Nov 27;25(23):12748. doi: 10.3390/ijms252312748 (PMC11641466; doi:10.3390/ijms252312748)
Supplement: Supplementary file 1 [file ijms-25-12748-s001.zip › Suplementary figures.pdf]

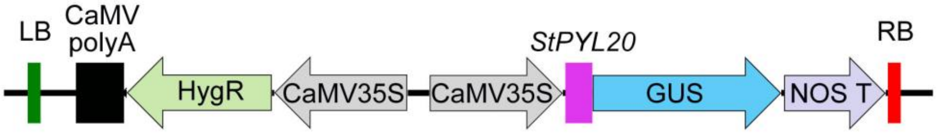

Fig. S1. Schematic diagram of the *StPYL20* gene plant-overexpression vector.

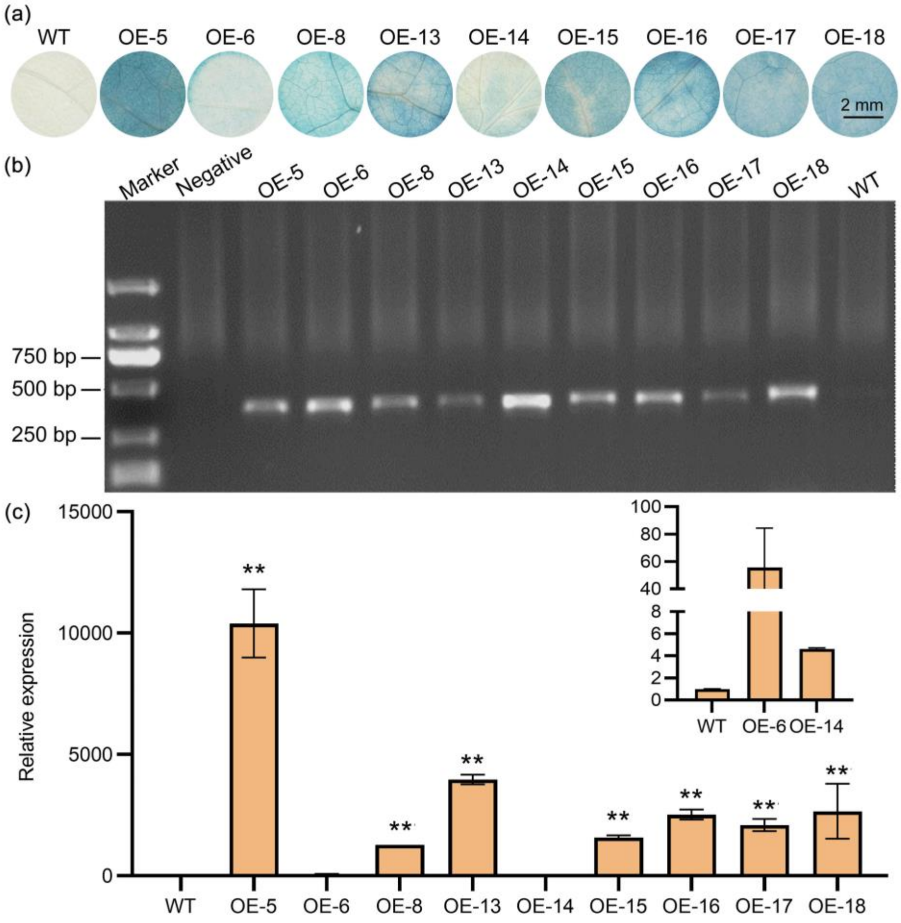

Fig. S2. Positive identification of *StPYL20* transgenic tobacco. (a) GUS histochemical staining of *StPYL20* transgenic lines and control plants; (b) PCR molecular identification of positive *StPYL20* transgenic lines; (c) Analysis of expression level of *StPYL20* in transgenic lines. ** represent significant differences between transgenic lines and WT at $P < 0.01$.

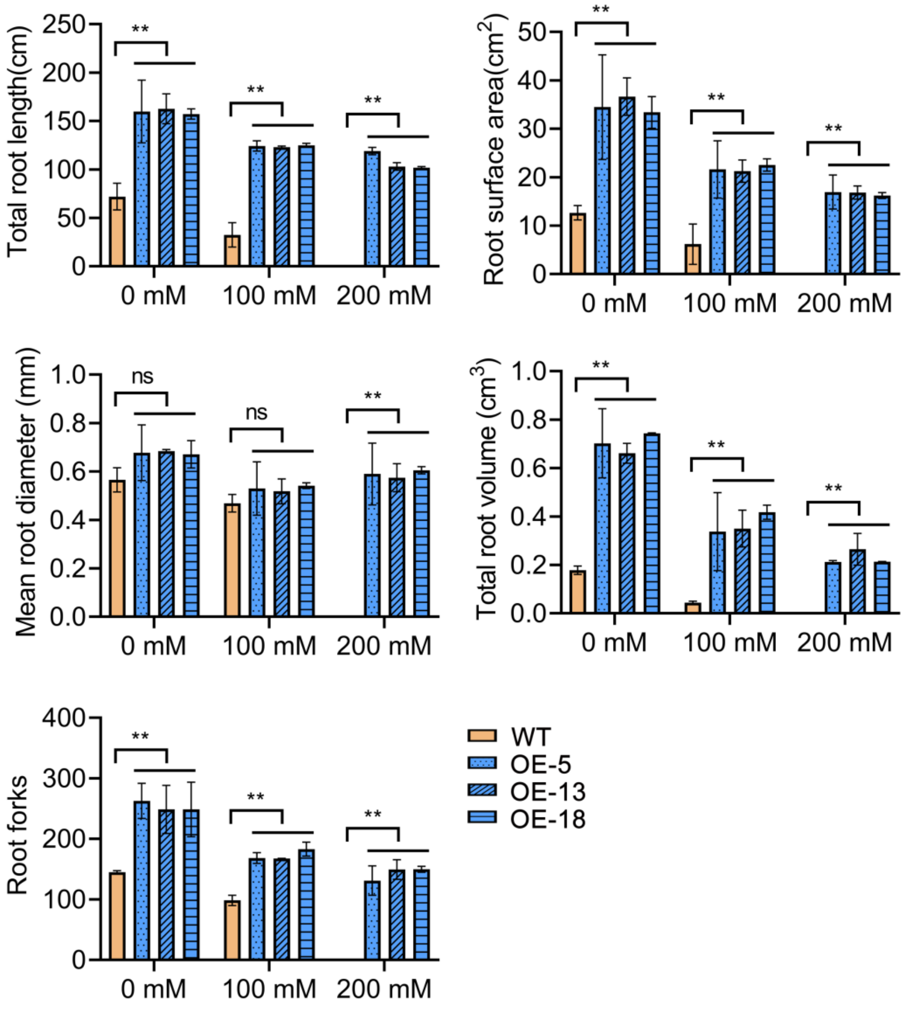

Fig. S3. Phenotypic data of roots for wild-type and overexpressing lines under mannitol stress conditions. 'ns' indicates no significant differences between the overexpressing lines and wild-type, while ** denotes a significant difference at the $P < 0.01$ level between the overexpressing lines and wild-type.

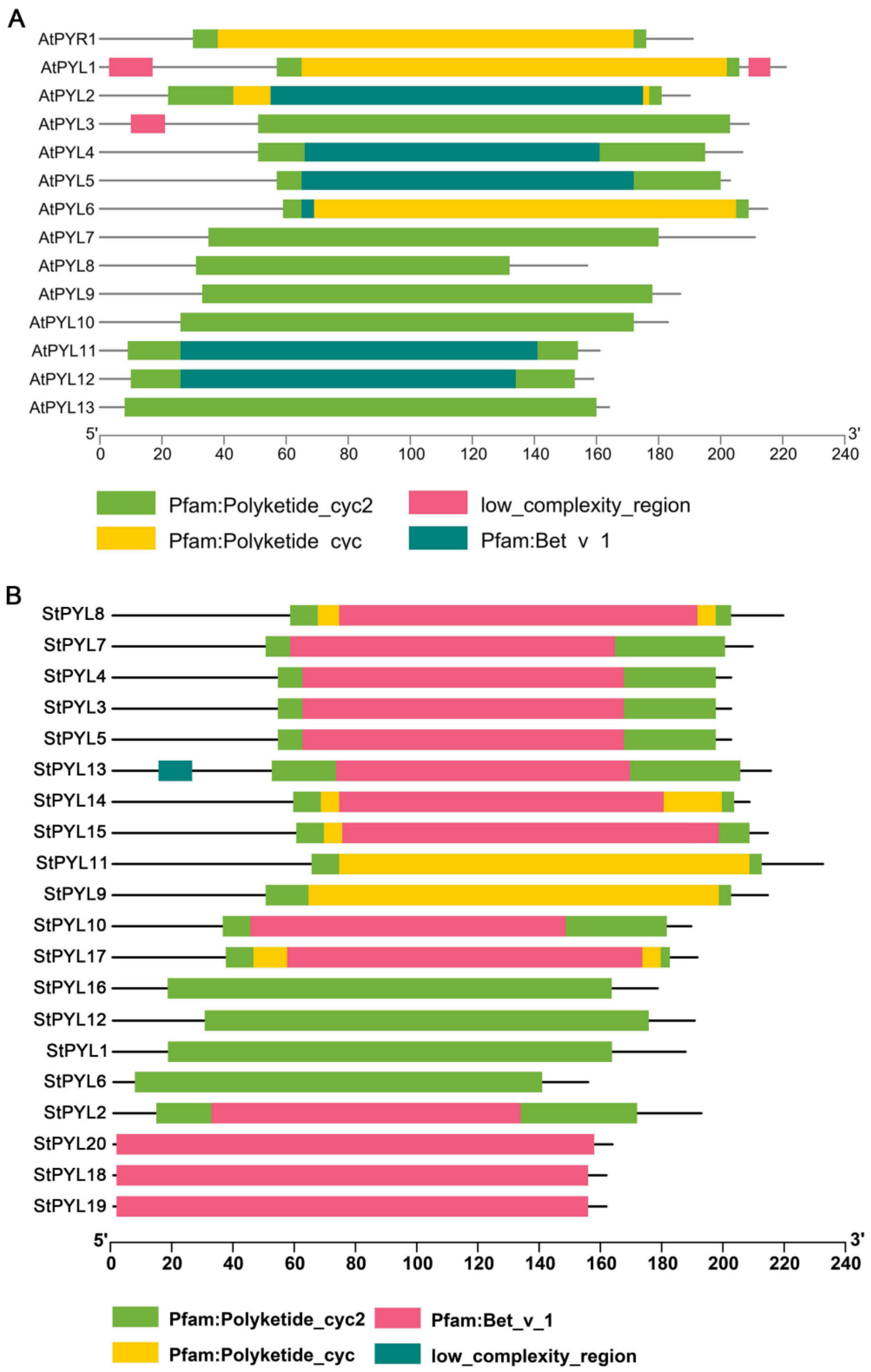

Fig. S4. Domains analysis of the PYL family in Arabidopsis (A) and potato (B).
